# Development of a genetic framework to improve the efficiency of bioactive delivery from blueberry

**DOI:** 10.1038/s41598-020-74280-w

**Published:** 2020-10-14

**Authors:** Molla F. Mengist, Haley Burtch, Hawi Debelo, Marti Pottorff, Hamed Bostan, Candace Nunn, Sydney Corbin, Colin D. Kay, Nahla Bassil, Kim Hummer, Mary Ann Lila, Mario G. Ferruzzi, Massimo Iorizzo

**Affiliations:** 1 Plants for Human Health Institute, North Carolina State University, 600 Laureate Way, Kannapolis, NC 28081 USA; 2 USDA-ARS-National Clonal Germplasm Repository, Corvallis, OR 97333 USA; 3 Department of Food Bioprocessing and Nutrition Sciences, North Carolina State University, Raleigh, 27606 NC USA; 4 Department of Horticultural Science, North Carolina State University, Raleigh, 27607 NC USA

**Keywords:** Metabolomics, Plant breeding, Secondary metabolism

## Abstract

In the present study, we applied a novel high-throughput in vitro gastrointestinal digestion model to phenotype bioaccessibility of phenolics in a diverse germplasm collection representing cultivated highbush blueberries. Results revealed significant (*P* < 0.05) differences between accessions, years, and accession by year interaction for relative and absolute bioaccessibility of flavonoids and phenolic acids. Broad sense heritability estimates revealed low to moderate inheritances of relative and absolute bioaccessibility, suggesting that besides environmental variables, genetics factors could control bioaccessibility of phenolics. Acylated anthocyanins had significantly higher relative bioaccessibility than non-acylated anthocyanins. Correlation analysis indicated that relative bioaccessibility did not show significant association with fruit quality or raw concentration of metabolites. The study also identified accessions that have high relative and absolute bioaccessibility values. Overall, combining the bioaccessibility of phenolics with genetic and genomic approaches will enable the identification of genotypes and genetic factors influencing these traits in blueberry.

## Introduction

The Dietary Guidelines for Americans has based recommendations for consumption of fruit and vegetables on evidence for their role in meeting nutrient needs and growing evidence of their association with chronic disease prevention^1^. While micronutrient content of fruits and vegetables remains critical to meeting nutrient needs, phytochemicals including phenolics present in many fruits have been associated with the prevention of several chronic and degenerative diseases^2^ which reinforces the rationale for recommended fruit intake levels. In particular, blueberry consumption has been associated with a reduced risk of diabetes^3–5^, cardiovascular disease^6,7^, cancer^8,9^, as well as Alzheimer’s disease and other neurodegenerative processes^10–12^. These benefits have been attributed to the diverse array and high concentration of phenolic compounds in blueberries including anthocyanins (ANC), phenolic acids (PA), flavonols (FLAV) and flavan-3-ols (F3L)^13–15^.

Blueberry belongs to the genus *Vaccinium*, which include multiple cultivated species (blueberry, cranberry, bilberry, lingonberries) that are of interest to consumers for their health properties. Three blueberry species, that include *V. corymbosum* L. (highbush blueberry, HB), *V. ashei* Reade (rabbiteye blueberry, RB) and *V. angustifolium* Ait (lowbush blueberry, LB), are commercially grown in the United States. While HB and rabbiteye blueberries are harvested from cultivated plantations, the lowbush blueberries, frequently termed “wild blueberries”, are gathered from native stands in Northeastern North America^16–18^. Among these species, the HB blueberry is widely grown across the US, accounting for ~ 95% of total blueberry production in the US^16,18,19^. The HB cultivars are further classified into northern highbush (NHB) and southern highbush (SHB) blueberries based on chilling requirement and winter hardiness^17^. Phenolic profiles have been assessed on a selected set of HB, and other wild and cultivated blueberry species to determine the diversity of phenolics within germplasm and to determine how these compounds relate to fruit quality and potentially to human health and nutrition^16,18,20–23^. These efforts have proven useful for breeding programs interested in understanding the genetic basis of phenolic synthesis and accumulation and to develop breeding strategies to enhance the phenolic content and potential health benefits of various blueberry species^18,24–26^.

However, in this context it is important to note that health benefits from consumption of phenolic-rich fruits may not only depend on the phenolic content and profiles in the fruit, but most critically on the efficiency of absorption and extent of metabolism (bioavailability) by the human body. In fact, enhancement of phenolic content in fruits does not always translate to higher bioavailability^27,28^. Maiz et al. (2016), reported that bioavailability of phenolics differed significantly between nine individual blueberry genotypes in a preclinical rodent model. Furthermore, these differences reflected the ability of genotypes with higher bioavailability to impact markers of bone health more so than low bioavailability genotypes^29,30^. The reason for these differences remains unclear but could be critical to understanding distinct bioactivity differences between these blueberry accessions.

While most current breeding programs seek to enhance our understanding of factors impacting blueberry phenolic profiles and content, little is known regarding traits that are associated with phenolic bioavailability from these fruits. One reason for this remains the notion that bioavailability is a trait best screened using costly and time-consuming in vivo models that can compare only small numbers of genotypically distinct fruits. As such, the potential to discover and ultimately leverage any existing variation in bioavailability as a genetically inherited trait remains untapped. The use of in vitro digestion models to estimate nutrient and phytochemical bioavailability from foods is well established^28,31–34^. Relying on predetermined human physiological conditions, these in vitro models typically measure bioaccessibility, the digestive release and solubilization of individual micronutrients or phytochemicals in the gut lumen^35,36^. Bioaccessibility is the amount of a compound that is released from the food matrix through normal digestion and made available for intestinal absorption^28,31,33,35,36^. Bioaccessibility estimates for phytochemicals have demonstrated good correlations and alignment with bioavailability in vivo^37–39^. Furthermore, these models have been instrumental in defining food matrix and processing factors that impact digestion and intestinal absorption of nutrients and phytochemicals including phenolics^32,40–43^. These models have been used to identify iron bioaccessibility as a genetically variable trait in maize^44^. Recently, variations in beta-carotene bioaccessibility were reported between 10 biofortified cassava genotypes including the variable influence of processing on this trait^45^. These efforts have served to guide breeding programs for the biofortification of crops targeting at-risk populations^31,45,46^. More recently, our group has successfully adapted this model to rapidly screen carotenoid bioaccessibility of 71 unique spinach genotypes, identifying a wide range in bioaccessibility and potentially high nutritive value genotypes^47^. These studies are increasing our understanding of the bioaccessibility of micronutrients and carotenoids. However, similar efforts have not been applied to study variation of phenolic bioaccessibility in high value fruits such as blueberry.

The main objective of the current study was to apply a semi-automated three-compartment in vitro gastrointestinal digestion model for high throughput (HT) phenotyping of the bioaccessibility of blueberry phenolics (ANC, PA, F3L, and FLAV). The resulting data were used to: (1) assess variability among the blueberry accessions for relative and absolute phenolic bioaccessibility; (2) investigate the association between bioaccessibility, phenolic content, and fruit quality traits [pH, titratable acidity (TA), total soluble solids (TSS) and fruit weight]; and (3) establish a strategy to study the genetic basis controlling bioaccessibility in highbush blueberry. The potential of leveraging this phenotyping method to characterize blueberry germplasm provides a unique opportunity to understand the nature of bioaccessibility as a new trait for application in plant genetic and breeding programs.

## Results and discussion

### In vitro digestion method development for flavonoids and phenolic acids

To quantify phenolics including ANC and non-ANC, commercially cultivated blueberries (unknown variety) were purchased at a local store in Kannapolis, NC (Supplementary Table S1). The composition was consistent with previous reports^18,20,21,48,49^. Among phenolics, ANC accounted for 283 mg/100 g FW within the form of simple glycosides (-3-*O*-galactoside (Galc), -3-*O*-glucoside (Gluc) and -3-*O*-arabinoside (Arab)) in relative abundance of malvidin (Mal) > petunidin (Pet) > cyanidin (Cyan) > peonidin (Peo) > delphinidin (Del). Acylated ANC accounted for < 10% of the total in the commercial blueberries. Chlorogenic acid (CHA) was the major PA found in commercial blueberries, with quercetin-3-glucoside (Q3Glu) and syringetin-3-glucoside (Syr3Glu) accounting for the majority of FLAV. Low levels of F3L epicatechin and catechin (0.3 and 0.2 mg/100 g FW) were also observed.

Bioaccessibility of phenolics using the commercial blueberries was first determined using both the low throughput (LT) and HT method to establish that the HT method adaptation by Hayes et al. (2020) would correlate well with the more established LT model. Relative and absolute bioaccessibility for individual phenolic species and by class (ANC, PA, FLAV, FL3) are presented in Table 1. With few exceptions, both relative and absolute bioaccessibility across individual phenolics and by sum of key classes were observed to be consistently higher in HT compared to LT methods. In general, both models were found to generate bioaccessibility values consistent with those previously reported for blueberry and other fruits^28,50,51^. While higher values were consistently observed with the HT method (Table 1), values obtained for the 24 targeted phenolic compounds between HT and LT models were determined to be highly correlated in both relative (r = 0.97) and absolute bioaccessibility (r = 0.98) (Supplementary Figure S1). Both models demonstrated high reproducibility and relatively low coefficient of variance (CV%) intra and inter-day (Table 1). Intra-day results showed that the HT model was slightly more consistent (CV; 1.3–19.5%, mean 7.5%) compared to the LT model (CV; 3.2–39.3%, mean 9.4%). However, the HT model appeared slightly more consistent inter-day (CV; 2.7–21.9%, mean 8.8%), compared to LT model (CV; 0.1–23.2%, mean 10.8%). Combined, these data suggested that the HT model was equally suitable for estimation of phenolic bioaccessibility as the more traditionally used LT model.

**Table 1:** Comparison of absolute (mg bioaccessible phenolics per 100 g blueberry material) and relative (%) bioaccessibility of individual phenolics from commercial blueberries determined by low throughput (LT) and high throughput (HT) models.

Model	LT		CV	HT		CV	p-value	p-value
Target	(Manual)		(%)	(Semi-Automated)		(%)	Absolute	Relative
**Anthocyanins (ANC)**								
Acylated_Del	5.6 ± 0.3	50%	9.80	6.4 ± 0.4	57%	6.40	0.01	0.01
Cyan3Galc	5.3 ± 0.3	25%	4.90	8.7 ± 0.4	40%	5.90	< .0001	< .0001
Cyan3Gluc	0.3 ± 0.0	33%	6.30	0.5 ± 0.1	49%	12.50	0.00	0.00
Cyan3Arab	3.6 ± 0.3	21%	7.90	6.4 ± 0.5	37%	9.50	< .0001	< .0001
Acylated_Cyan	2.6 ± 0.5	49%	20.40	2.6 ± 0.5	48%	21.90	0.84	0.93
Peo3Galc	7.1 ± 0.3	43%	4.80	10.7 ± 0.4	63%	3.30	< .0001	< .0001
Peo3Gluc	0.7 ± 0.0	49%	4.10	0.8 ± 0.1	53%	13.50	0.07	0.23
Peo3Arab	4.9 ± 0.1	43%	1.70	7.7 ± 0.2	66%	1.30	< .0001	< .0001
Pet3Galc	2.2 ± 0.2	6%	1.40	4.4 ± 0.4	12%	8.20	< .0001	< .0001
Pet3Gluc	0.1 ± 0.0	6%	16.20	0.1 ± 0.0	12%	4.60	0.33	< .0001
Pet3Arab	1.6 ± 0.1	6%	4.80	3.1 ± 0.3	13%	6.00	< .0001	< .0001
Acylated_Pet	3.4 ± 0.6	62%	16.10	3.8 ± 0.4	68%	10.70	0.12	0.12
Mal3Galc	21.5 ± 1.2	36%	5.70	32.0 ± 1.1	52%	2.50	< .0001	< .0001
Mal3Gluc	1.0 ± 0.2	47%	22.90	1.6 ± 0.1	75%	8.20	< .0001	< .0001
Mal3Arab	18.2 ± 0.8	41%	4.70	27.0 ± 0.7	59%	1.30	< .0001	< .0001
Acylated_Mal	0.4 ± 0.1	49%	13.50	0.6 ± 0.1	69%	8.20	< .0001	0.00
Total glycosides	66.4 ± 1.6	30%	7.80	103.0 ± 1.5	44%	6.50	< .0001	< .0001
Total_Acylated_ANC	16.7 ± 2.8	53%	14.90	17.9 ± 1.3	61%	11.80	0.23	0.24
TotalANC	83.1 ± 4.2	35%	9.60	121.0 ± 2.8	49%	7.80	< .0001	0.01
**Phenolic acid (PA)**								
CHA	27.8 ± 5.6	33%	20.20	33.3 ± 3.5	39%	11.30	0.03	0.05
Vanillic Acid	0.2 ± 0.0	71%	7.10	0.2 ± 0.0	72%	15.60	0.37	0.87
Caffeic Acid	0.8 ± 0.2	231%	20.60	0.9 ± 0.1	255%	13.60	0.15	0.25
PA	28.7 ± 5.8	112%	16.00	34.4 ± 3.6	122%	13.50	0.10	0.49
*Flavanol (FLAV)*								
Q3Gluc	17.2 ± 1.7	42%	7.60	18.4 ± 1.8	48%	9.30	0.17	0.01
Q3Arab	1.9 ± 0.1	39%	10.80	1.9 ± 0.2	46%	7.30	0.93	0.01
Syr3Gluc	6.1 ± 0.6	49%	10.70	6.9 ± 0.7	56%	9.80	0.02	0.02
FLAV	25.2 ± 2.4	44%	9.70	27.2 ± 2.6	50%	8.80	0.03	0.21
**Flava-3-ol(F3L)**								
Catechin	0.1 ± 0.0	28%	7.70	0.1 ± 0.0	37%	10.40	0.15	0.00
Epicatechin	0.1 ± 0.0	20%	23.20	0.1 ± 0.0	22%	12.80	0.41	0.27
F3L	0.1 ± 0.0	24%	15.50	0.1 ± 0.0	29%	11.60	0.03	0.03

Values represent mean ± standard deviation of n = 4 replicates digested per day for a total of 3 days. A total n = 12 digestions per sample are represented.

P-values (< 0.05) denote a significant difference in bioaccessibility between models (LT v. HT).

Anthocyanidins abbreviation: Cyan, cyanidin; Del, delphinidin; Mal, malvidin; Peo, peonidin; Pet, petunidin. Sugar moieties abbreviation: Arab, arabinoside; Galc, galactoside; Gluc, glucoside. Others: ac, acylated; syr, syringetin; Q, quercetin; CHA, chlorogenic acid.

### Distribution and variation in in vitro bioaccessibility of flavonoids and phenolic acids in blueberry accessions

Results of phenolic concentration in raw blueberry samples selected from the USDA National Clonal Germplasm Repository (NCGR) germplasm and their bioaccessibility are presented in Supplementary Table S2. Combined analysis of variance revealed significant (*P* < 0.05) effects for accession, year, and accession by year interaction for raw concentration of all phenolics (Supplementary Table S3). The 66 blueberry accessions exhibited considerable phenotypic variation for raw concentrations of phenolics. For the 2017 data, the raw concentration of ANC, PA, FLAV and F3L exhibited a 9, 114, 12 and 4 fold-change (maximum value/minimum value), respectively. Similar levels of variations were also observed for the 2018 data. For both years, ANC were identified as the most abundant phenolic compounds in blueberry fruit followed by PA, whereas F3L were the least abundant metabolites (Supplementary Table S2, Supplementary Figure S2A). These results are consistent with other studies^18,20,48,52,53^. The presence of significant genotypic effects controlling phenolic metabolite concentration and fruit quality traits in blueberry has been reported^16,18,22,23,54,55^.

Relative bioaccessibility of flavonoids and PA was significantly (*P* < 0.05) influenced by accession, year and accession by year interaction (Supplementary Table S3). The variation in relative bioaccessibility among the accessions for total ANC, PA, FLAV and F3L ranged from 10–40%, 3–64%, 37–100%, and 2–96%, respectively (Supplementary Table S2). Examination of the absolute bioaccessibility revealed significant (*P* < 0.05) effects due to accession, year, accession by year (Supplementary Table S3). Among accessions, the bioaccessible content for total ANC, PA, FLAV and F3L, ranged from 4, 96, 12 and 94-fold change, respectively (Supplementary Table S2). While the inheritance of bioaccessibility has not yet been evaluated, a few studies have reported the presence of varietal differences in bioaccessibility or bioavailability of phenolics^27,56,57^. However, it is important to note that bioaccessibility is a surrogate of bioavailability and is used as an indicator of food or matrix factors that impact bioavailability.

**Figure 1:**
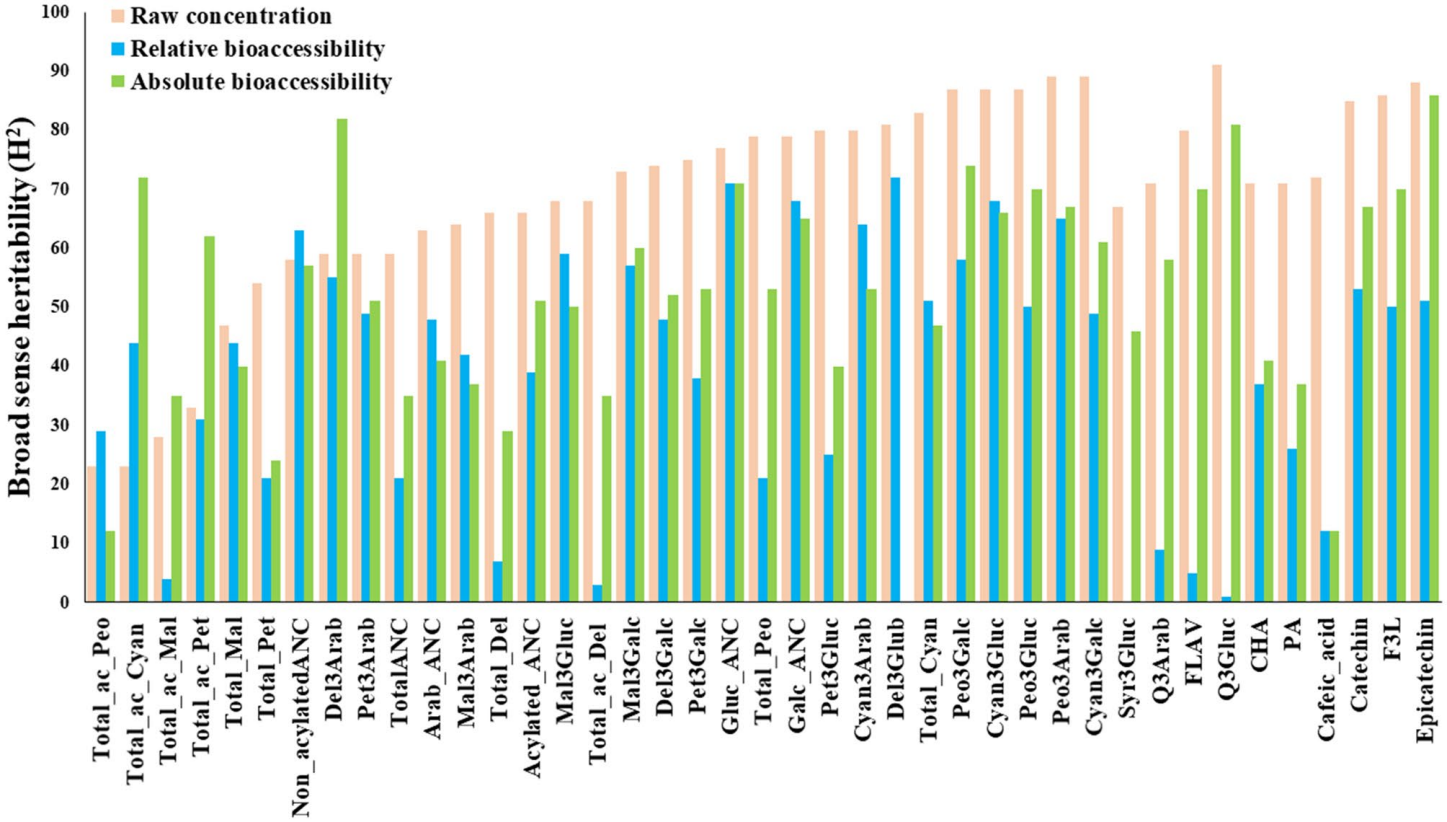
Broad sense heritability estimates for raw concentrations, relative and absolute bioaccessibility traits in 66 blueberry accessions over two years. Anthocyanidins abbreviation: Cyan, cyanidin; Del, delphinidin; Mal, malvidin; Peo, peonidin; Pet, petunidin. Sugar moieties abbreviation: Arab, arabinoside; Galc, galactoside; Gluc, glucoside. Others: ac, acylated; syr, syringetin; Q, quercetin.

Broad sense heritability estimates were performed to estimate the contribution of genetic factors regulating relative and absolute bioaccessibility of all metabolites evaluated (Fig. 1). Broad sense heritability ranged from 20 to 90% for raw concentration of the metabolites evaluated here. However, the relative bioaccessibility of most of the phenolics revealed a low to moderate (< 50%) broad sense heritability (Fig. 1), suggesting that the relative bioaccessibility of the metabolites evaluated is highly influenced by environmental factors and/or in vitro digestion conditions. However, the relative bioaccessibility of some phenolics, such as non-acylated ANC, Gluc_ANC, Total_Cyan, Cyan_3_Gluc and Peo_3_Gluc exhibited more than 50% broad sense heritability (Fig. 1). The broad sense heritability of the absolute bioaccessibility tended to have a similar pattern to the broad sense heritability of raw metabolites concentration (Fig. 1). Results of broad sense heritability estimates were consistent with our previous findings^18^, that raw concentration of phenolic metabolites and fruit quality traits such as ANC, F3L, TSS and fruit weight have moderate to high (> 50%) broad sense heritability. This is the first study that estimates broad sense heritability of flavonoid and PA relative and absolute bioaccessibility in blueberry. Given the extensive variation among accessions for raw concentrations and in vitro digestibility together with considerable heritable traits, it may be possible to study genes contributing to improve bioaccessibility of phenolic metabolites including ANC, PA, FLAV and F3L. This information could be used to select parental lines for crossing and screening of breeding materials. From long term perspective, this information could further be used to design cost effective DNA assays to use in breeding programs for selecting new blueberry cultivars with improved bioaccessibility.

We also compared the relative and absolute bioaccessibility among the metabolite classes. On average, FLAV (63%) and F3L (50%) had higher relative bioaccessibility than total ANC (23%) and PA (26%) (Supplementary Table S2, Supplementary Figure 2B,C). While, ANC (1081 mg/100 g FW) and PA (583 mg/100 g FW) had higher bioaccessible content than FLAV (238 mg/100 g FW) and F3L (53 mg/100 g FW). Other studies have reported variation in terms of in vitro digestion recovery between metabolites^28,49,52,53,58,59^. In blackberry, a higher relative bioaccessibility was recorded for quercetin derivatives (40–80%) followed by PA (27.5%); whereas, ANC were the least recovery metabolite (< 10%)^59^. These results are consistent with the results observed in this study. While the reason for this variation remains to be explored, it could be related to a combination of relative concentrations and/or individual stability of phenolics to digestive conditions within each metabolite class that contributes to the observed differences.

**Figure 2:**
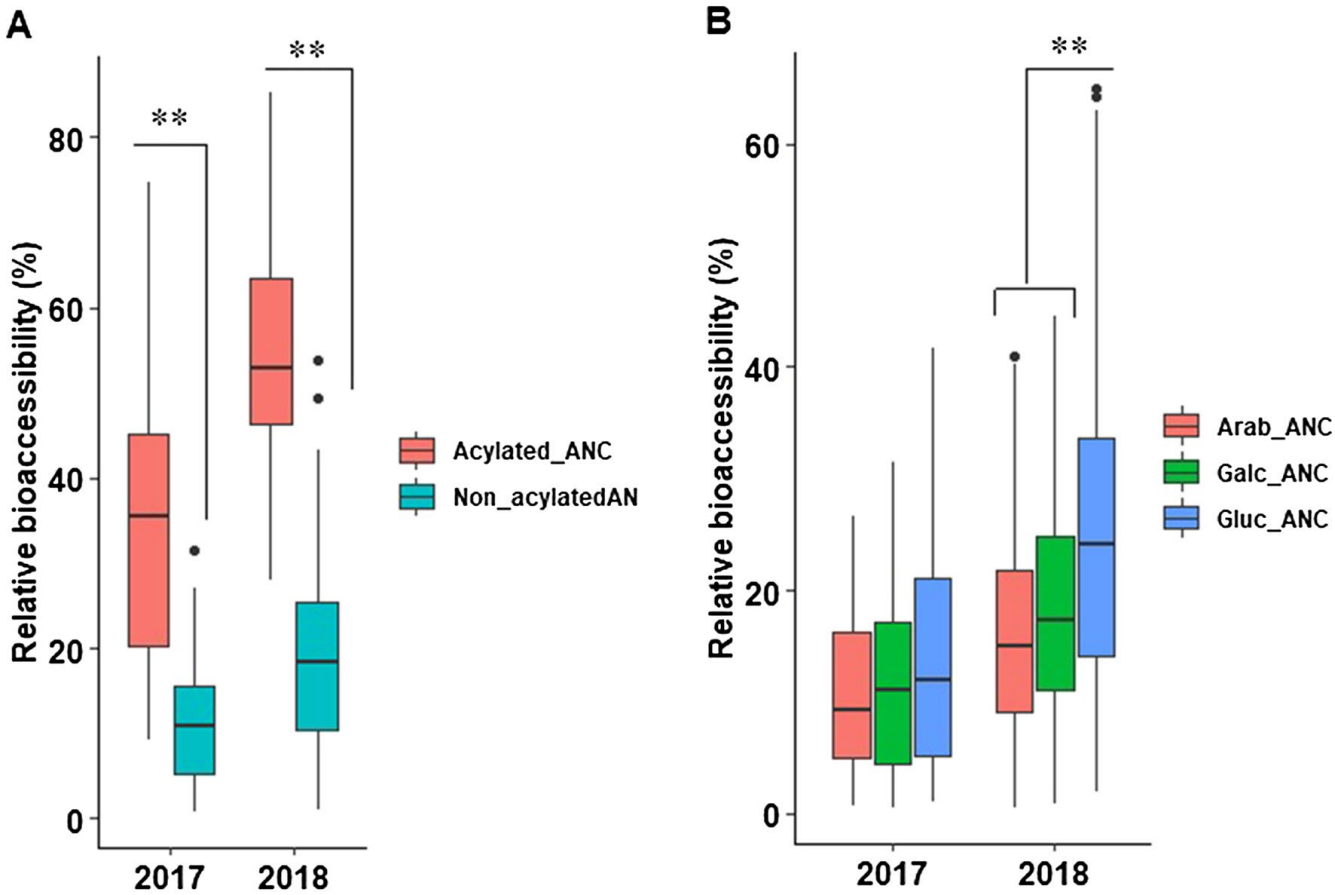
Boxplot showing variation in relative bioaccessibility anthocyanins. (**A**) acylation, (**B**) sugar moieties. Relative bioaccessibility of the trait; Arab, arabinoside; Galc, galactoside; Gluc, glucoside. **, statistically significant at P < 0.01.

We examined the relative bioaccessibility of individual FLAV and F3L compounds and found that there were no significant differences in relative bioaccessibility between the different FLAV for 2017 data. However, significant (*P* < 0.01) differences were observed for 2018 data, FLAV glucoside had a higher relative bioaccessibility than arabinoside containing FLAV (Supplementary Figure S3). The two F3L compounds, catechin and epicatechin, also differed in relative bioaccessibility for the 2018 data; catechin had a higher relative bioaccessibility than epicatechin for both years (Supplementary Figure S3).

### Effects of acylation, types of sugar moieties and aglycone on in vitro bioaccessibility of anthocyanins in blueberry

Comparison between acylated and non-acylated ANC revealed that acylated ANC had significantly (*P* < 0.01) higher relative bioaccessibility than non-acylated ANC for both years (Fig. 2A), suggesting that the acylation of ANC may contribute to the increased bioaccessibility of ANC in vitro. Studies regarding relative bioaccessibility of ANC are limited; however, a few studies observed that acylation of ANC may increase the bioaccessibility of ANC in wild blueberries and red cabbage^49,60^. This effect could be due to a higher stability of acylated ANC to gastrointestinal conditions of elevated pH^49,60^. Although no study has reported the importance of acylation for the bioavailability of phenolics from blueberry, there is evidence in other crops, such as sweet potato, red wine^61^ and red cabbage^62^ that acylated ANC have higher bioavailability than non-acylated ANC. While direct translation of bioaccessibility data to in vivo bioavailability remains complicated, the higher bioaccessibility observed here suggests that this could be a factor contributing to the higher observed bioavailability for acylated ANC in vivo.

For sugar moieties, glucoside containing ANC had statistically significant (*P* < 0.01) higher relative bioaccessibility than galactoside or arabinoside containing ANC for the 2018 season. While a similar trend was observed for 2017 data, this did not reach statistical significance (Fig. 2B). Currently, no study has compared the impact of glycosylation moiety on the bioaccessibility or bioavailability of ANC in blueberries; however, some studies have reported that glycosylated ANC are more bioavailable in grapes, wines and natural food pigments^51,63,64^.

We also compared the relative bioaccessibility of metabolites based on their non-glycosylated aglycone structures (anthocyanidins) and found that ANC with malvidin (hydroxy-dimethoxy backbone) or peonidin (hydroxy-methoxy backbone) aglycones had higher relative bioaccessibility than cyanidin (dihydroxy) or petunidin (dihydroxy-methoxy) containing aglycones (Supplementary Figure S4). No direct evidence is available regarding the effect of anthocyanidin structure on the bioaccessibility or bioavailability of ANC. However, cyanidin and delphinidin (trihydroxy backbone) are less stable chemically and more susceptible to auto-oxidative reactions by virtue of their catechol and galloyl B ring structure. As such, they may oxidize during digestion more rapidly compared to methylated malvidin and others^65^.

### Association between in vitro bioaccessibility of flavonoids and phenolic acids and fruit quality traits in blueberry accessions

**Figure 3:**
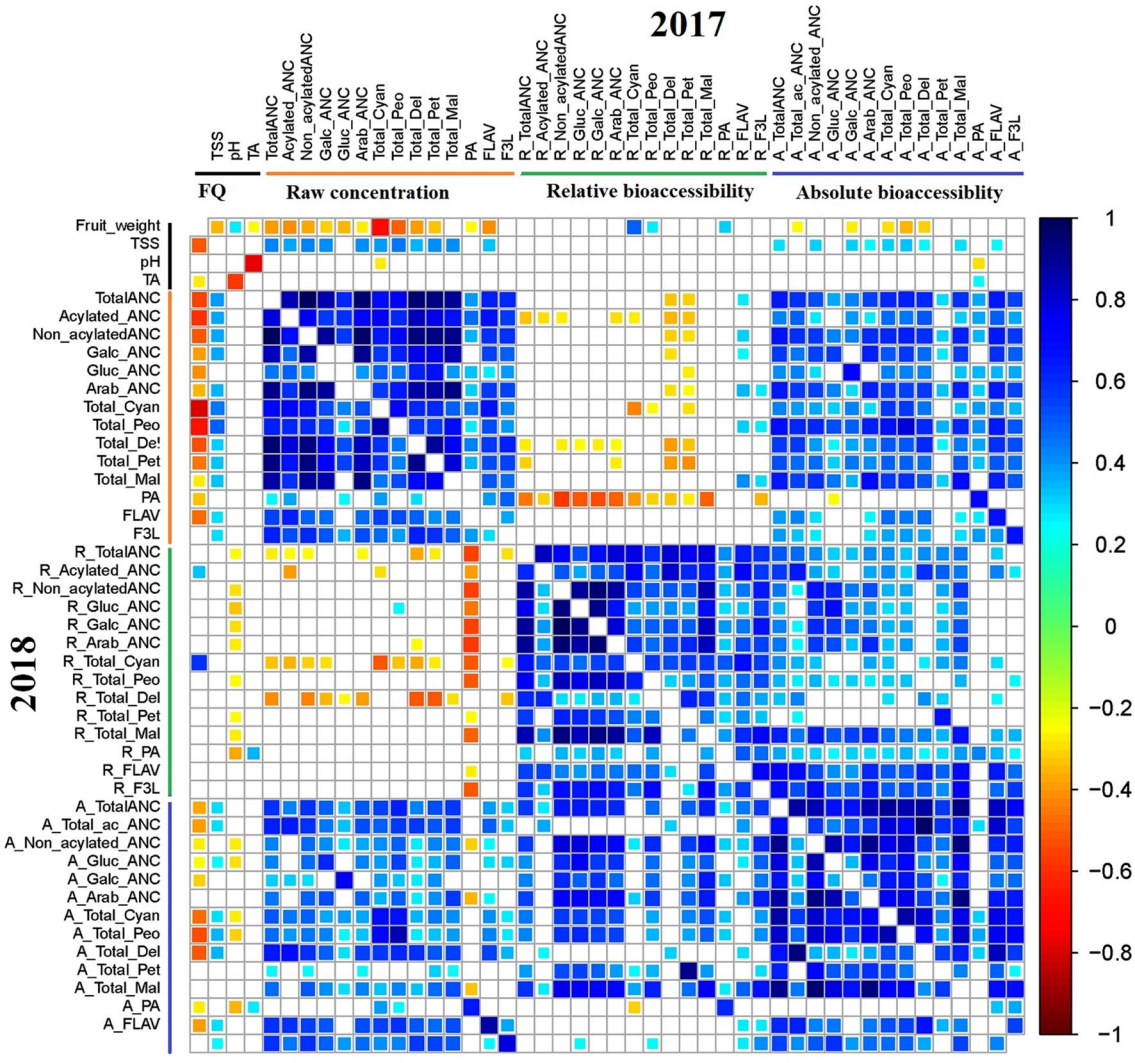
Correlation analysis of raw concentration, relative and absolute bioaccessibility of phenolics in 66 blueberry accessions over two years. The color bar indicates metabolite classes and fruit quality traits. Cyan-to-blue and yellow-to-red colors show significant (P < 0.05) positive and negative correlation between traits, respectively.

Pearson coefficient of correlation analysis between metabolite, and fruit quality traits was performed for the two years of data independently (Fig. 3). For the 2017 data, fruit weight was negatively correlated with the raw concentration and absolute bioaccessibility of ANC, F3L, and PA. As expected, TSS was positively correlated with flavonoids (ANC, F3L and FLAV). However, fruit weight and TSS did not show any significant correlation with relative bioaccessibility. Relative bioaccessibility of most of the metabolites did not show significant correlation with their respective raw concentration. As expected, both relative bioaccessibility and raw concentration of most of the metabolites exhibited a significant positive correlation with their respective absolute bioaccessibility. For the 2018 data, the correlation patterns were similar to that obtained for the 2017 data except for pH, which was negatively correlated with relative bioaccessibility of most metabolites (Fig. 3). Another observation was that PA was negatively correlated with relative bioaccessibility of most metabolites for both years (Fig. 3). Previous studies have reported a positive association between flavonoids and TSS^18,23,54,55^. This is not surprising since TSS is mainly composed of sugar molecules, and sugar molecules are the major structural component of flavonoid glycosides. In addition, negative associations of blueberry fruit size with the raw concentrations of most metabolites have been previously reported^18,23,54,55^. Anthocyanins are predominantly found in the skin of blueberry. Assuming uniform skin thick and inverse relationship between fruit size and fruit surface area, it is expected that smaller fruit have higher surface area, thereby given the same amount of material, smaller sized fruit will have higher anthocyanin content as compared to larger sized fruits^18,55^. For both years, TA was positively associated with the relative bioaccessibility of phenolic acids. As expected, higher TA was observed at low pH (acidic) condition for both years (Fig. 3). The acidic condition of digestion may help to facilitate the release of polyphenols from the plant matrix^66,67^.

We acknowledge that other fruit characteristics such as texture could affect bioaccessibility. Indeed, the release of phenolic compounds from the fruit matrix is favored by the mechanical disruption. Furthermore, the cell wall components such as cellulose, hemicellulose and pectin could interact with polyphenols, and influence their bioaccessibility^66,67^. Hence, we plan to address this question in future work.

**Figure 4:**
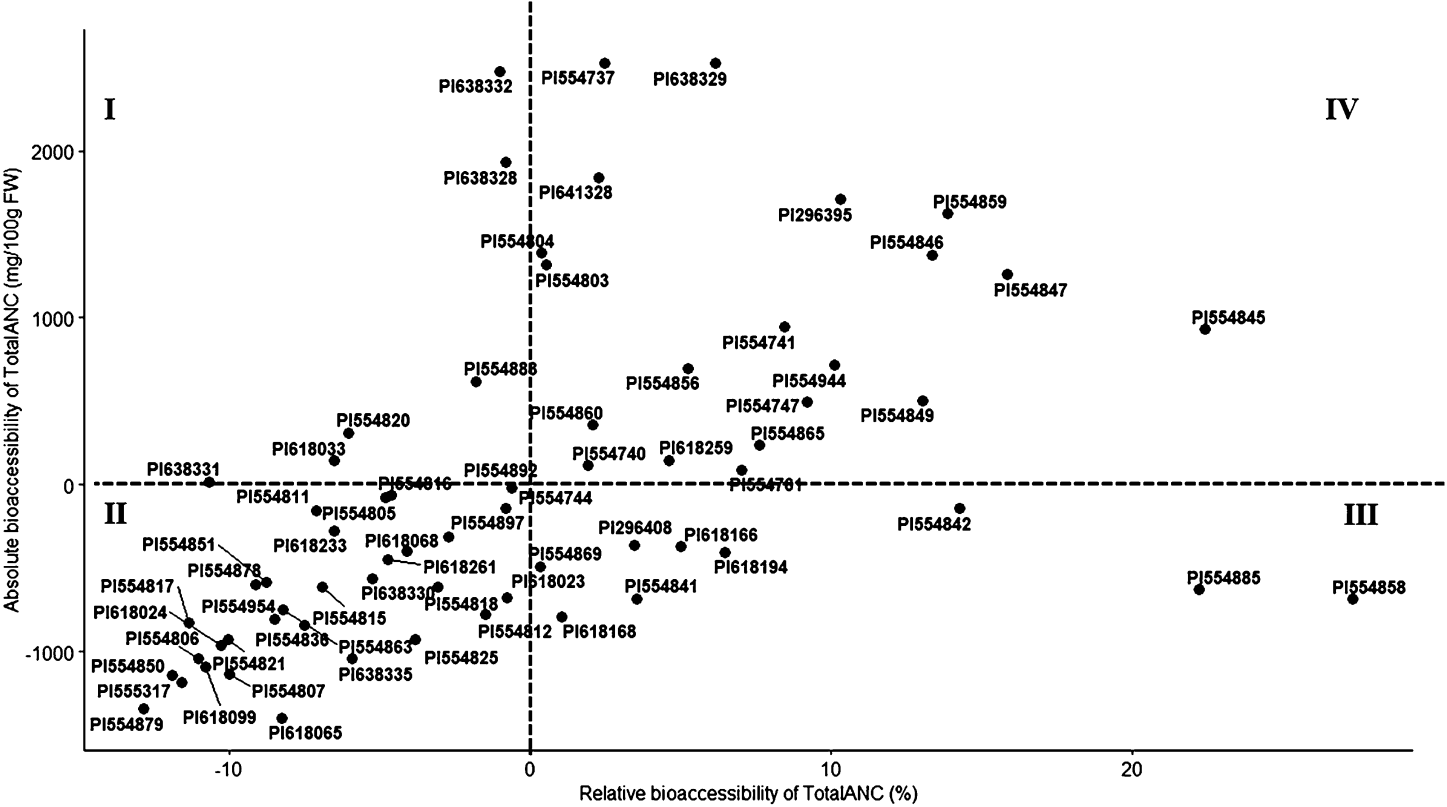
Correlation between relative and absolute bioaccessibility of total anthocyanin in 66 blueberry accessions. The relative and absolute bioaccessibility of total anthocyanin for 66 blueberry accessions was compared with the grand mean, relative bioaccessibility (17.32%) and absolute bioaccessibility (1674 mg/100 g FW). Each of the four quadrants indicate: high absolute bioaccessibility (> 1674 mg/100 g FW) and low relative bioaccessibility (< 17.32%) (I); low relative bioaccessibility (< 17.32%) and low absolute bioaccessibility (< 1674 mg/100 g FW)(II); low absolute bioaccessibility (< 1674 mg/100 g FW) and high relative bioaccessibility (> 17.32%) (III), and high absolute bioaccessibility (> 1674 mg/100 g FW) and high relative bioaccessibility (> 17.32%) (IV).

Given the positive correlation between relative and absolute bioaccessibility of Total ANC (Fig. 4), a scatterplot was generated to identify accessions that have higher than average relative and absolute bioaccessibility. A total of 20 (30%) accessions had scored above the average for both relative and absolute bioaccessibility of Total ANC, suggesting that there is room to improve both relative and absolute bioaccessibility of ANC simultaneously in blueberry breeding programs.

### Multivariate analysis of in vitro bioaccessibility of flavonoids and PA and fruit quality traits

**Figure 5:**
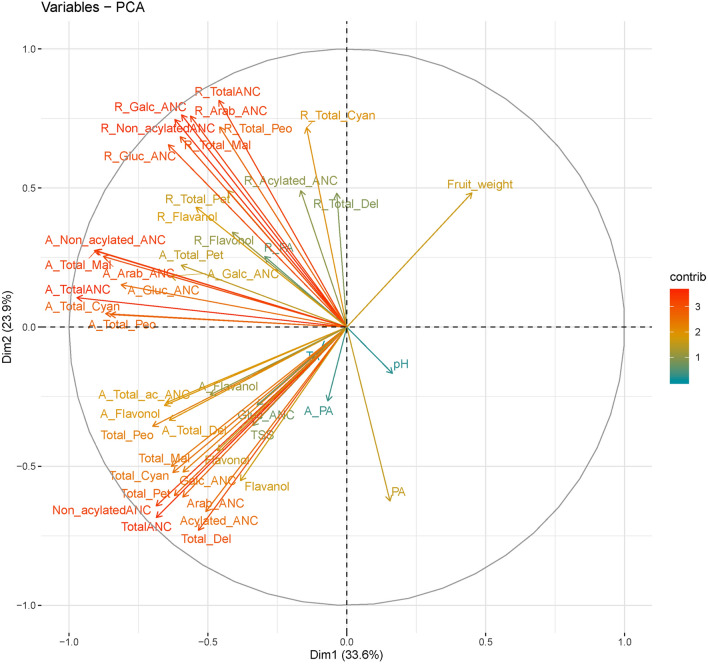
PCA loading scores of the first and second principal components of relative and absolute bioaccessibility of phenolics and fruit quality data from 66 blueberry accessions.

The scree plot of the PCA displays the variance explained for each component (Supplementary Figure S5C). Accordingly, the first two principal components (PC) accounted for 33.6% and 23.9% of the variance, respectively (Fig. 5; Supplementary Figure S5C). To identify the key traits discriminating the different accessions, loading scores of the first two PC were examined (Fig. 5) and raw concentration, relative and absolute bioaccessibility, and fruit quality data with highest loading scores on PC1/2 were identified for all 66 accessions (Fig. 5). Accordingly, absolute accessibility traits including A_TotalANC, A_non-acylated ANC, A_Total_Mal and A_Arab revealed the highest loading score in PC1 (Supplementary Figure S5A). However, relative bioaccessibility traits such as R_TotalANC, R_Galc_ANC, R_Arab ANC and R_non_acylated ANC contributed to PC2 (Supplementary Figure S5B). Overall, absolute and relative bioaccessibility significantly contributed to the first and second PC, respectively (Fig. 5). A PCA biplot depicted the relationships between accessions and variables. Accessions such as PI554845, PI618259 and PI618068 had high values for relative bioaccessibility, whereas, PI554865, PI554803 and PI554741 had high scores for absolute bioaccessibility (Supplementary Figure S6). In addition, we assessed whether metabolite profiles and the respective bioaccessibility data could discriminate accessions based on types of highbush blueberry (NHB, SHB and their hybrids). Results from the PCA indicated the absence of distinctive bioaccessibility features between the highbush blueberry and their hybrids (Supplementary Figure S7). These results are consistent with previous studies that NHB and SHB did not show distinctive features as evaluated by molecular markers or phenolic metabolite content^18,68,69^. The two highbush blueberry classifications, NHB and SHB, are commonly used in blueberry breeding programs for the improvement of agronomic and bioactive metabolite traits^24,70^.

Finally, the application of high-throughput genotyping platforms and statistical models enabled plant geneticists and breeders to dissect the genetic basis of important agronomic and nutritional traits. As a result, advanced molecular tools are becoming available for breeding programs to accelerate the selection of new improved cultivars and pyramid multiple traits including the relatively untapped trait of bioactive and micronutrient bioavailability. This study applied a high-throughput phenotyping method to evaluate in vitro bioaccessibility of flavonoids and PA in blueberry. Application of the method demonstrated notable differences among blueberry accessions for in vitro bioaccessibility of flavonoids and PA evaluated here. Unlike absolute bioaccessibility, relative bioaccessibility did not correlate with the raw metabolite concentrations, suggesting that it is not possible to predict in vitro bioaccessibility based on the raw concentrations. Hence, simultaneous selection of cultivars with high raw concentration and high relative bioaccessibility would result in higher bioaccessible content of flavonoids and PA. Overall, there appeared to be significant phenotypic variations and moderate broad sense heritability for bioaccessibility of phenolics evaluated here. The study also highlights that acylation increases relative bioaccessibility of ANC. Finally, fruit quality traits such as fruit size, TA and TSS do not show any association with relative bioaccessibility. These findings combined with continuous expansion and improvement in bioaccessibility models to include lower gastrointestinal (GI) digestion and metabolism provide a framework for development of varieties through phenotypic selection and/or using molecular markers with greater impacts on human health. One limitation of this study was that bioaccessibility screening was conducted on homogenized samples only. This likely resulted in normalization of additional genotypic variation based on textural or other rheological properties. While done to simplify the high throughput screening, it is possible that phenolic bioaccessibility from fruits can differ between whole fruit and pureed or juiced samples^71^. As such future efforts to include oral processing in future assessments is warranted.

## Materials and methods

### In vitro digestion method development for flavonoids and PA for blueberry

#### Chemicals, solutions, and standards

Chromatography solvents (acetonitrile, methanol, water and formic acid) and phenolic standards (cyanidin 3-*O*-glucoside, delphinidin 3-*O*-glucoside, peonidin 3-*O*-glucoside, petunidin 3-*O*-glucoside, malvidin 3-*O*-glucoside, catechin, epicatechin, caffeic acid, vanillic acid, chlorogenic acid, syringetin 3-*O*-glucoside and quercetin 3-*O*-glucoside) were obtained from Sigma Aldrich (St. Louis, MO, USA). Internal standards, ethyl gallate and taxifolin, were obtained from TCI (Portland, OR, USA) and Selleckchem (Houston, TX, USA), respectively. Materials required for the in vitro digestion including mucin (M2378), α-amylase (A3176), pepsin (P7125), lipase (L3126), pancreatin (P7545), and bile (B8631) were obtained from Sigma Aldrich, while urea (U15-500), and uric acid (A13346-14) were purchased from Fisher Chemical (Fair Lawn, NJ, USA). The oral phase solution for the in vitro digestions was prepared as described^40^ by combination of KCl, Na_3_PO_4_, Na_2_SO_4_, NaCl, and NaHCO_3_ in distilled water at concentrations of 242, 11, 8, 10, and 40 mmol/L, respectively.

#### Blueberry material and processing for in vitro digestion

Experiments to adapt and validate the HT in vitro digestion method were conducted using commercial cultivated blueberries obtained from a local market in Kannapolis, NC. Frozen blueberries (85-100 g) were thawed for approximately 30 min and coarsely blended (Waring Commercial Laboratory) for 2 min followed by a more complete homogenization using a VWR 250 homogenizer equipped with a VWR 20 mm × 200 mm Saw-Tooth Generator Probe for 1.5 min to generate a smooth puree-like consistency. Once processed, commercial blueberry purees were aliquoted and stored at − 80 °C until use as technical replicates in validation experiments for the in vitro digestion methods.

#### Manual low throughput (LT) in vitro gastrointestinal digestion

The phenolic bioaccessibility of blueberries was first assessed with a previously reported static three-phase in vitro digestion model as described^32^ with slight modifications. Briefly, ~ 3.0 g of processed blueberry homogenate (or puree) was aliquoted to a 50 mL Falcon tube and the oral phase was initiated by adding 6 mL of oral phase solution containing 31 mg/mL of α-amylase. Samples were vortexed, blanketed with N_2_ and placed in an oscillating incubator (37 °C, 85 opm, 10 min). Following the oral phase, the gastric phase of digestion was initiated by diluting samples to 30 mL with 0.9% saline and the addition of porcine pepsin solution (2 mL, 20 mg/mL in 0.1 M HCl). Sample pH was adjusted to pH 2.5 ± 0.1 with 1.0 M HCl and the gastric volume was brought to 40 mL with saline solution. Samples were vortexed, blanketed with N_2_ and incubated at 37 °C (85 opm, 1 h). Following incubation, the small intestinal phase was initiated by neutralization of gastric acid with 1.0 M NaHCO_3_ and adjustment of the pH 6.5 ± 0.1. Small intestinal enzymes (2 mL, 20 mg/mL pancreatin, 10 mg/mL lipase in 0.1 M NaHCO_3_) and porcine bile salts (3 mL, 30 mg/mL bile extract in 0.1 M NaHCO_3_) were added to samples prior to standardization to a final volume of 50 mL with saline. Samples were again vortexed, blanketed with N_2_ and incubated (37 °C, 85 opm, 2 h). Following the intestinal phase, the final crude digested material known as digesta was centrifuged (10,400×*g*, 4 °C, 1 h) to isolate the aqueous bioaccessible fraction (AQ). The AQ was collected, then filtered through a 0.22 µm filter (Macherery Nagel, Bethlehem, PA, USA) and stored at − 80 °C until further analysis.

#### Semi-automated high throughput (HT) in vitro gastrointestinal digestion

Adaptation of the standard in vitro digestion model to accommodate higher throughput has been described^47^. Briefly, successful adaptation to the standard model required manipulation of model parameters including proportional reductions in starting material and total digestion volume, modified mixing conditions, filtering enzyme solutions to avoid particulate, as well as integrating the adjusted model with an automated fluid-handling robot (Tecan EVO 150, Tecan; Mannedorf, Switzerland). Utilizing the Tecan as opposed to manual execution promoted increased speed while providing more precise reagent distribution and sample transfers, enhanced throughput and walkaway time. Aliquots (1.1 g/15 mL reaction tube) of processed blueberries were placed into Tecan sample racks (16 tubes/rack) and loaded into defined Tecan columns. Reaction tubes were subjected to a simulated 1.8 mL oral phase solution containing α-amylase that had been previously centrifuged at 10,600×*g* for 10 min to remove any potential particulates that may disrupt aspiration of the fine-tipped Tecan syringes. Tube racks were removed from the Tecan robot and reaction tubes were individually vortexed, placed back within racks and blanketed with N_2_. Tube racks were then stacked into a closed, oscillating incubator (37 °C, 155 opm, 10 min). Once removed from the incubator, reactions were diluted with 5.7 mL of 0.9% saline solution and introduced to the gastric phase by the addition of 0.6 mL porcine pepsin solution (10 mg/mL in 0.1 M HCl). A control sample (wild blueberry freeze-dried powder) and a random blueberry sample were selected and removed from each Tecan rack and adjusted to a pH of 2.5 ± 0.1 with 1.0 M HCl. Control samples were used to ensure consistency of enzyme solution preparation and Tecan function between separate runs. HCl addition to randomly selected blueberry samples was averaged, determining the appropriate amount of the 1.0 M HCl for application to the entire blueberry subset. Following pH adjustment, 0.9% saline solution was added to sample tubes bringing the volume total to 12 mL prior to blanketing with N_2_ and incubating (37 °C, 155 OPM, 1 h). The intestinal phase was initiated by the sequential addition of a combination of 0.6 mL small intestinal enzymes (20 mg/mL of pancreatin and 10 mg/mL of lipase in 0.1 M NaHCO_3_ centrifuged at 10,400×*g* rpm for 10 min) and 0.9 mL porcine bile salts (30 mg/mL bile extract in 0.1 M NaHCO_3_). Reactions were adjusted to pH 6.5 ± 0.1 with 1 M NaOH_3_, brought up to a digestion volume of 15 mL with 0.9% saline solution, blanketed with N_2_ and incubated (37 °C, 155 OPM, 2 h). Following intestinal incubation, racks were immediately removed from the incubator and placed back in the Tecan where the robot aspirated a total of 3 mL of the digesta (DG), from each reaction tube and dispensed 1.5 mL into 2 separate 96-well (2 mL) collection plates (Waters US, Milford, MA, USA) consecutively; the second plate was intended as a back-up. Plates were blanketed with N_2_ and covered with 96-well square plug silicone/polytetrafluoroethylene cap-mats (Waters US, Milford, MA, USA) and secured within the centrifuge (2,988×*g*, 1 h). Post-centrifugation, the 96-well plates were immediately placed on ice where the separated AQ fraction was removed, filtered through a 0.20 µm cellulose acetate filter (Macherery Nagel, Bethlehem, PA, USA) and stored in a -80 °C freezer until further analysis.

#### Phenolic extraction and analysis by LC–MS

Phenolics were extracted from processed blueberry by solid phase extraction using a method adapted from^32^ and^72^. Briefly, 500 mg of blueberry homogenate was combined with 5 mL of 2% formic acid in 80% methanol vortexed for ~ 1 min and then centrifuged at 3600×*g* for 4 min. The supernatant was collected, and the extraction was repeated 3 times. The combined supernatants were dried under N_2_ and resolubilized in 5 mL of 0.1% formic acid in 50:50 methanol and water and loaded a polymeric reversed-phase, 96-well plate (Phenomenex, StrataX 33 µm, 10 mg/well). Loading volume were ~ 1000 µL per sample (methanolic extract from blueberry homogenate diluted in 1% formic acid in water) or approximately 1000 µL aqueous fraction (filtered aqueous fraction diluted in 1% formic acid in water). All samples were spiked prior to the extraction with 5 µL ethyl gallate (200 µM) for determination of extraction recovery. Solid phase extraction (SPE) plates were rinsed with 2% formic acid in water. Elution of targeted phenolics was completed with 0.1% formic acid in methanol and collected into a 96-round well, polypropylene 350 µL collection plate (Waters, Milford MA). 10 µL of taxifolin (200 µM) was added as a volume control to adjust for any variation in volume between wells following SPE. Extraction recovery from blueberry fruit and aqueous digesta fractions was found to range from 79–109% with average recovery of 96 ± 6%. Blueberry phenolics were subsequently characterized by LC–MS using a method adapted^73^. Samples were injected into a Waters H-Class ACQUITY *UPLC* equipped with an ACQUITY *UPLC* BEH C18 column (1.7 µm, 2.1 mm × 150 mm). Phenolic compounds were separated with a gradient elution using a binary mobile phase composed by 0.1% formic acid in acetonitrile as solvent A and 2% formic acid in water as solvent B. Following separation, individual phenolics were detected using a Waters QDA mass selective detector by means of individual single ion response (SIR) in both negative (phenolics and flavonoids) and positive ion mode (ANC). A total of 24 phenolic compounds representative of blueberry were selected and targeted for analysis (Supplementary Table S4). Each blueberry homogenate and /or aqueous fraction resulting from digestion was analyzed once by LC. Authentic standards of each compound, with limited exception, were used to determine existing compounds within samples and were further used to quantify such compounds via calibration curves developed from individual SIR (Supplementary Table S4). Final concentrations were adjusted by each individual sample extraction recovery as determined by ethyl gallate internal standard response and taxifolin volume control. Concentrations of all ANC compounds were based on the response of the -3-O-glucoside form of each parent compound, while other glycosides (galactoside, arabinoside, acylated derivatives). Individual anthocyanin glycosides were tentatively identified elution order assigned as -3-O-Galactoside, -3-O-Glucoside and -3-O-Arabinoside based on previous validation in our laboratory and comparison to previous reports from our group and others of this elution order under similar chromatographic conditions^20,74,75^. Individual anthocyanins were quantified using the response of the parent -3-O-glucoside SIR as previously described^73^*.*The matrix effect factor (MEF) for blueberry fruit and aqueous digesta were calculated from experiments using post extraction addition of taxifolin as an internal standard. An MEF of 11.7 ± 3.1 and 5.0 ± 1.6 for blueberry fruit and aqueous digesta extract was observed. While this may not be reflective of broader matrix effects impacting each blueberry phenolic analyte, this level of MEF is suggestive of a similar but modest to minor ion suppression effect between extracts as determined by taxifolin internal standard response.

#### Estimation of relative and absolute bioaccessibility

Relative (%) bioaccessibility was defined as the percentage of polyphenols from the blueberry raw material recovered in the aqueous digesta (AQ) after simulated digestion. Absolute bioaccessibility (mg of bioaccessible phenolics per 100 g fresh weight (FW) blueberry) is derived by multiplying the relative (%) bioaccessibility and the phenolic content in a 100 g FW serving of the starting blueberry material. Bioaccessibility of blueberry phenolics is expressed both by individual phenolic compounds and as sum of phenolics within each class including ANC, PA, FLAV and F3L to improve presentation clarity and facilitate class comparisons and analysis.

### Application of the method for evaluating variability between blueberry accessions

#### Materials collection and preparation

A total of 66 tetraploid blueberry accessions were obtained from the NCGR, Corvallis, OR, United States. Detailed descriptions of the accessions are provided in Supplementary Table S5. For each accession, berries were harvested at the ripe fruit stage from two clonal plants for two consecutive years, 2017 and 2018. The amount of fruit available from each clone was not the same and, in several cases, not sufficient to perform all of the phenotyping assays^18^. Therefore, prior to processing, fruits were combined per genotype and then separated into three technical replicates. The technical replicates should minimize errors associated with sample processing and fruit quality and metabolite trait phenotyping^18^. After harvesting, the berries were stored at − 80 ℃, shipped on dry ice to the Plants for Human Health Institute (PHHI), Kannapolis, North Carolina, United States, and stored at − 80 ℃ until processing. Frozen berries (approximately 10–30 g, three replicates), were then used for fruit quality and metabolite analyses.

#### Phenotyping of fruit quality and in vitro bioaccessibility of flavonoids and phenolic acids

Fruit quality traits including fruit weight, pH, TA and TSS were evaluated as described^18^. Briefly, fruit weight (g per fruit) was recorded (10–30 berries total for three technical replicates), and TSS , pH and TA were assessed for fruit harvested in 2017 and 2018. The berries used to measure fruit weight were homogenized into a puree using a Waring Commercial Blender 7012G (Torrington, CT, United States). Homogenized samples were used to determine TSS, pH and TA. TSS was measured using a digital hand-held “pocket” refractometer PAL-1 (Atago, Tokyo, Japan) and the results were expressed as ◦Brix. The pH and TA were measured using 1 g of homogenized sample diluted with 30 ml pre-boiled double distilled water. The pH was measured using an Accumet AB15, pH-meter (Fisher Scientific, Waltham, MA, United States). Then, TA was determined with a Mettler DL15 Auto-Titrator (Columbus, OH, United States) at pH 8.2 using 0.02 mol L^−1^ sodium hydroxide. TA was expressed as percentage of citric acid (wt/wt) per 1 g FW^18^. Metabolite extraction, quantification, in vitro digestion and bioaccessibility estimates on the 66 accessions, were performed as described above using the semi-automated HT model (see 2.1.4).

#### Analysis of variance, trait heritability, and correlation analyses

To assess the magnitude of variation between accessions, we computed a minimum, maximum and range of variation for all metabolites and fruit quality traits. Fold-change values were calculated independently for each metabolite and fruit quality trait, dividing the maximum value by the minimum value of each trait. Analysis of variance (ANOVA) was performed to partition individual metabolite traits according to accession, year, and accession by year interaction^18,76^. Best linear unbiased estimate (BLUE) data obtained from the linear model were used as the phenotypic values for clustering and principal component analysis (PCA) analysis^18,76^. Broad sense heritability (*H*^2^) was estimated using variance components calculated from the restricted maximum likelihood (REML)^77^, calculated as follows:

$${H}^{2}=\frac{{\delta }_{g}^{2}}{{(\delta }_{g}^{2}+\frac{{\delta }_{gy}^{2}}{y}+\frac{{\delta }_{e }^{2}}{ry})}$$

where ${\delta }_{g}^{2}$,${\delta }_{gy}^{2}$ and ${\delta }_{e }^{2}$ are variance components of accessions, [genotype x environment] interaction, and residual variations, respectively; y is the number of environments (number of years in this study, 2) and r is the number of replications (r = 3). Pearson Coefficient of Correlation was performed to find the relationship among traits and for the two-year data, independently. The correlation was visualized using the R package corrplot^78^.

#### Multivariate analysis of metabolites and fruit quality traits

BLUE data obtained from linear effects model were used as an input file for PCA. PCA is a technique used to reduce dimensionality of the data by finding linear combinations (dimensions; in this case, the number of metabolite and fruit quality traits) of the data. PCA was performed using the R package Fact Miner^79^ as a non-supervised method to identify key traits with the largest effect on the overall variability and to evaluate the effect of genetic background on fruit quality and metabolite profiles among different accessions.

## Supplementary information

Supplementary Figures.

Supplementary Tables.
