# Supplementary material for: Development of a genetic framework to improve the efficiency of bioactive delivery from blueberry
Source: Sci Rep. 2020 Oct 14;10:17311. doi: 10.1038/s41598-020-74280-w (PMC7560831; doi:10.1038/s41598-020-74280-w)
Supplement: Supplementary file 1 — Supplementary Figures. [file 41598_2020_74280_MOESM1_ESM.docx]

**Development of a genetic framework to improve the efficiency of bioactive delivery from blueberry**

Molla F. Mengist^1^, Haley Burtch^1^, Hawi Debelo^1^, Marti Pottorff^1^, Hamed Bostan^1^, Candace Nunn^1^, Sydney Corbin^1^, Colin D. Kay^1,3^, Nahla Bassil^2^, Kim Hummer^2^, Mary Ann Lila^1,3^, Mario G. Ferruzzi^1,3*^, Massimo Iorizzo^1, 4*^

^1^ Plants for Human Health Institute, North Carolina State University, 600 Laureate Way, Kannapolis, NC, 28081, USA

^2^ USDA-ARS-National Clonal Germplasm Repository, Corvallis, OR 97333, USA

^3^ Department of Food Bioprocessing and Nutrition Sciences, North Carolina State University, Raleigh, North Carolina, USA

^4^ Department of Horticultural Science, North Carolina State University, Raleigh, North Carolina, USA

*Correspondence:

Corresponding Authors: mferruz@ncsu.edu; miorizz@ncsu.edu


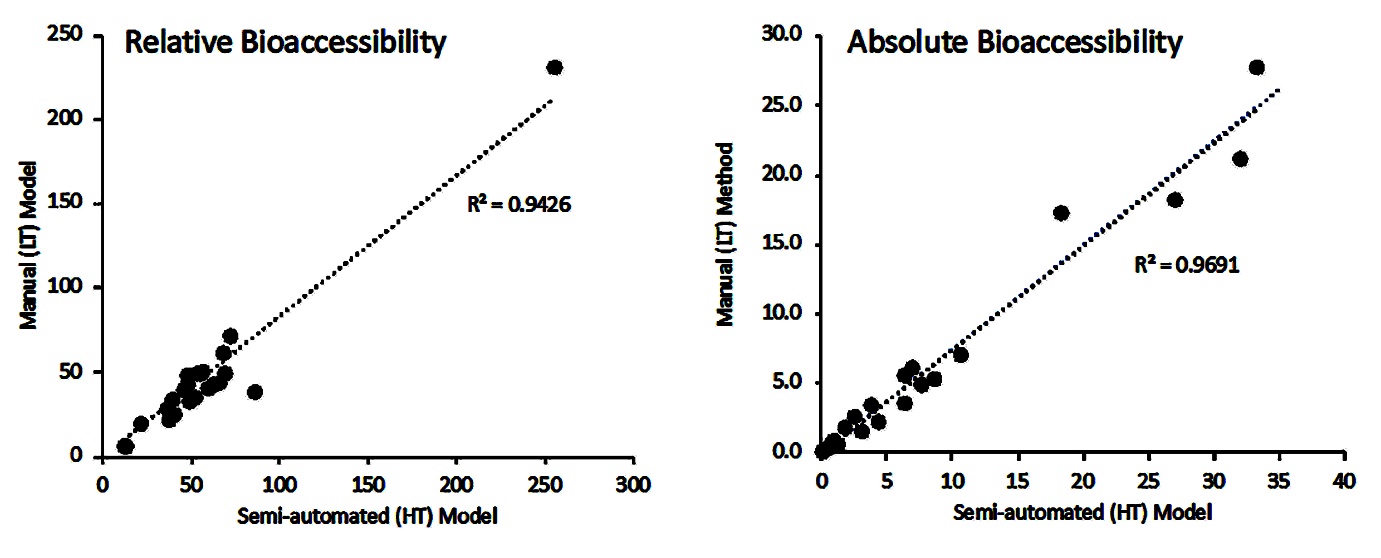


**Supplemental Figure S1 Correlation between *in vitro* digestion models Low Technology (LT) vs. High Technology (HT) in terms of average inter-day bioaccessibility (relative (%) vs. absolute (mg/100 g FW). Circular markers indicate all targeted phenolic compounds (Supplementary Table S1) within the broader phenolic classes (ANC, PA, FLAV, F3L).**


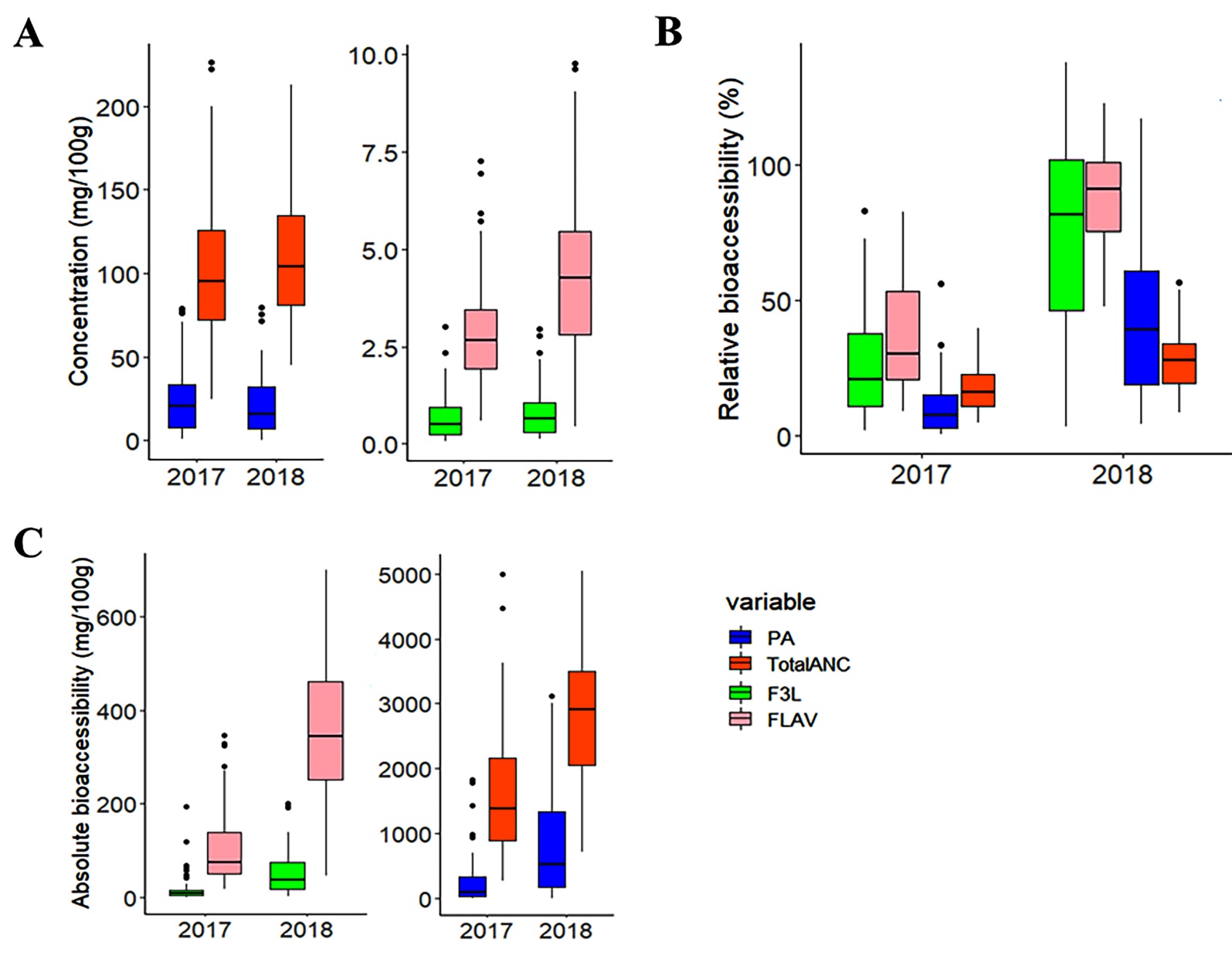


Supplementary Figure S2. Boxplot showing variations in metabolite concentration, relative and absolute bioaccessibility of phenolics for 66 blueberry accessions over two years, 2017 and 2018. (A) Variation in raw metabolite concentrations, (B) variation in relative bioaccessibility, and (C) variation in absolute bioaccessibility.


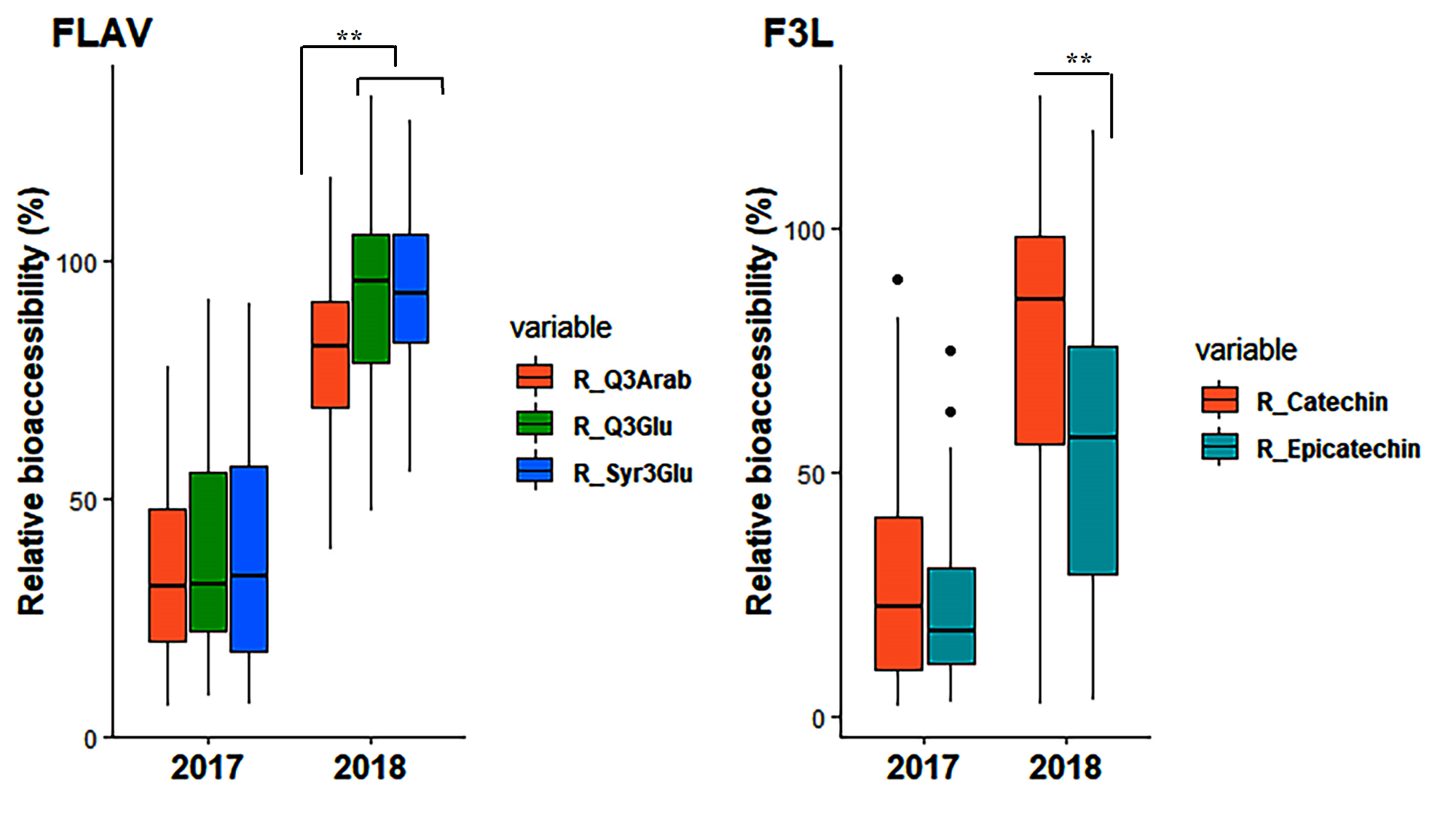


Supplementary Figure S3. Boxplot showing variation in relative bioaccessibility for flavanol and flavonol from 66 blueberry accessions phenotyped over two years, 2017 and 2018. Abbreviations: R_, relative bioaccessibility; Q3arab, quercetin-3-arabinoside; Q3Glu, quercetin-3-glucoside; Syr3Gluc, Syringetin-3-glucoside. **, statistically significant at *P<0.01*.


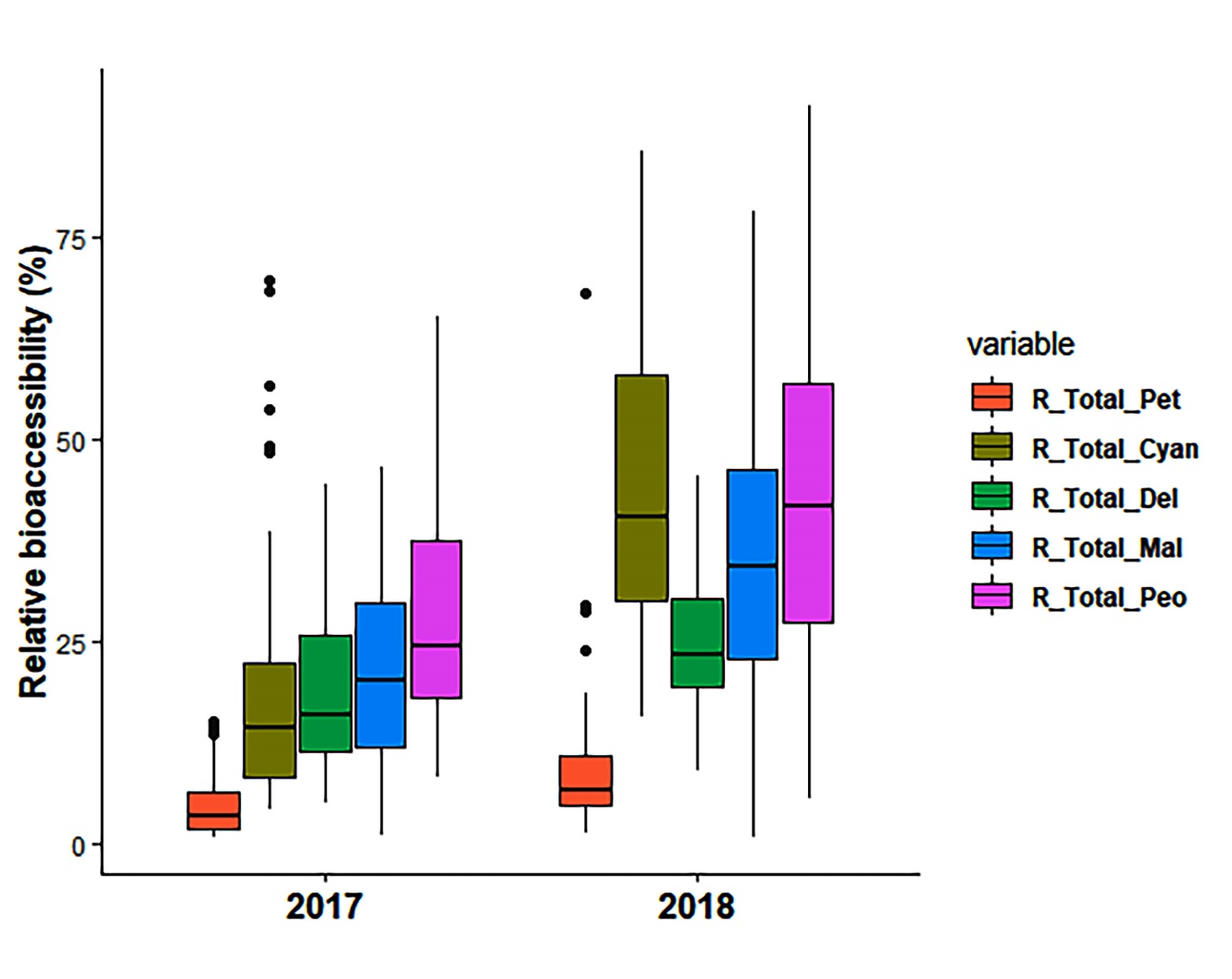


Supplementary Figure S4. Boxplot showing variation in relative bioaccessibility between the different anthocyanidins from 66 blueberry accessions phenotyped over two years, 2017 and 2018. Abbreviations: Cyan, cyanidin; Peo, peonidin; Del, delphinidin; Mal, malvidin; Pet, petunidin.


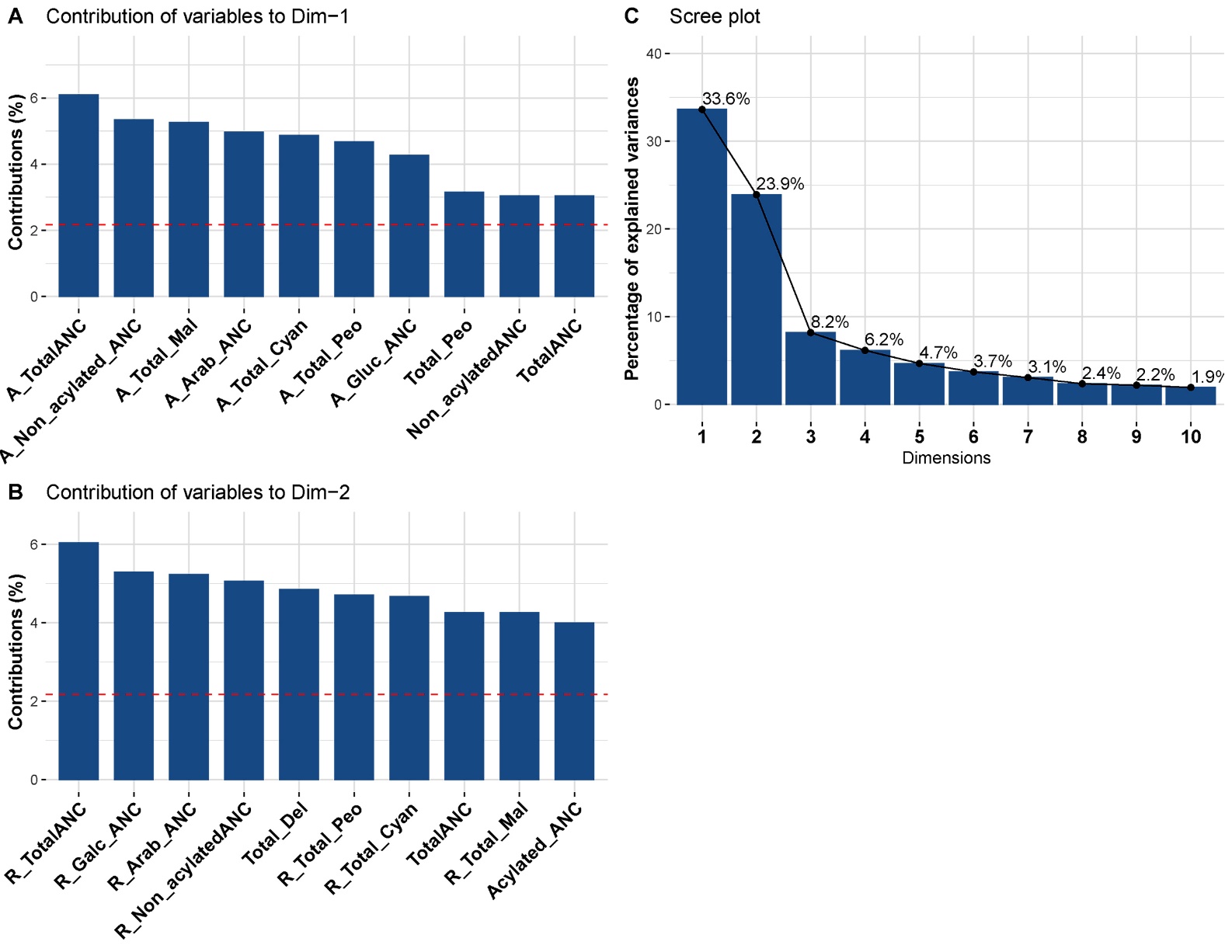


Supplementary Figure S5. Variable contributions to the first two components and PCA scree plot (PC1-PC10) of bioaccessibility and fruit quality data from 66 blueberry accessions. Variable contribution (%) to the first (A), second (B) principal components of the PCA; the relative variation explained by each principal component (C).


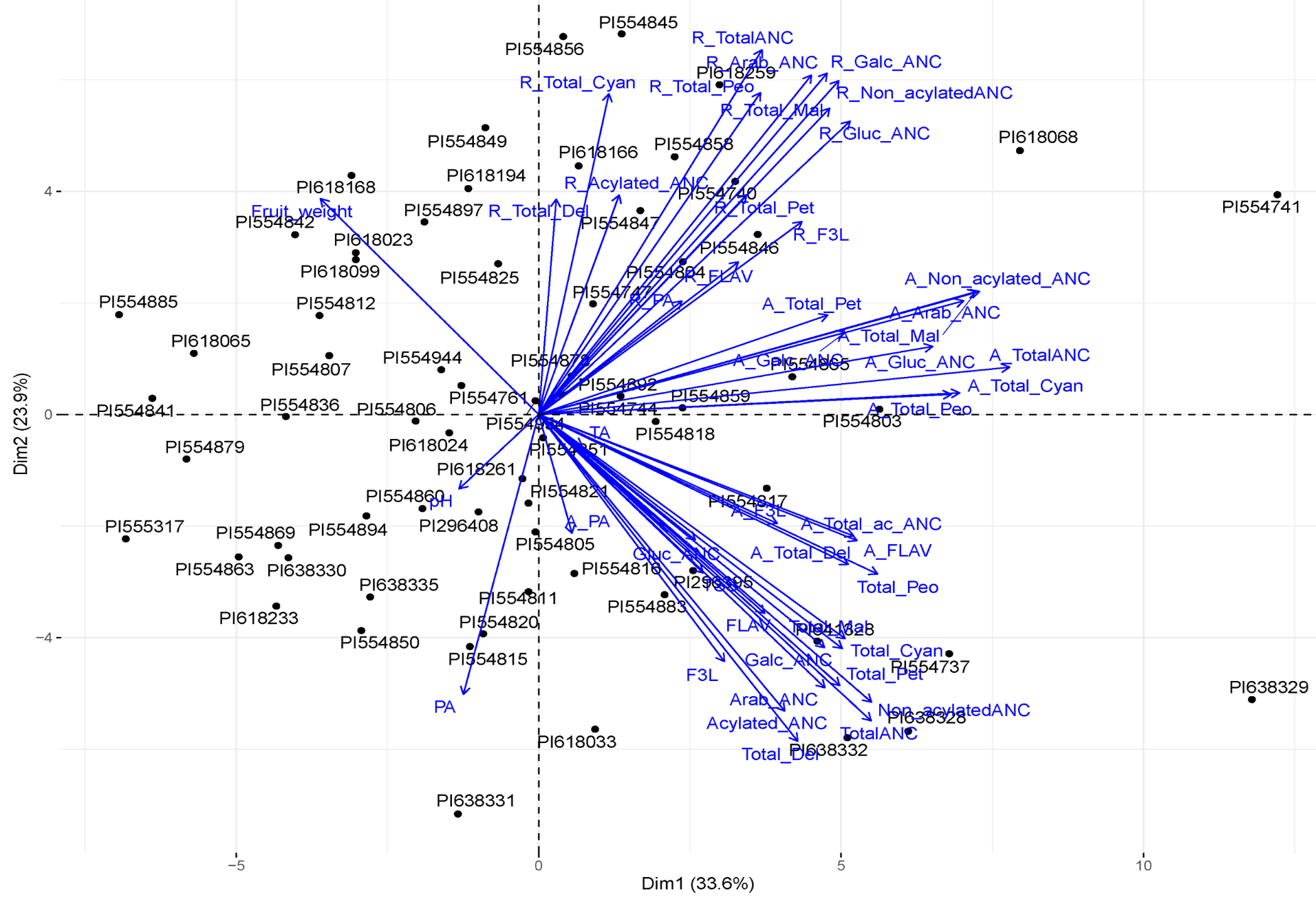


Supplementary Figure S6. PCA biplot showing the affiliation between variables and individual accessions in the first two PCA plots.


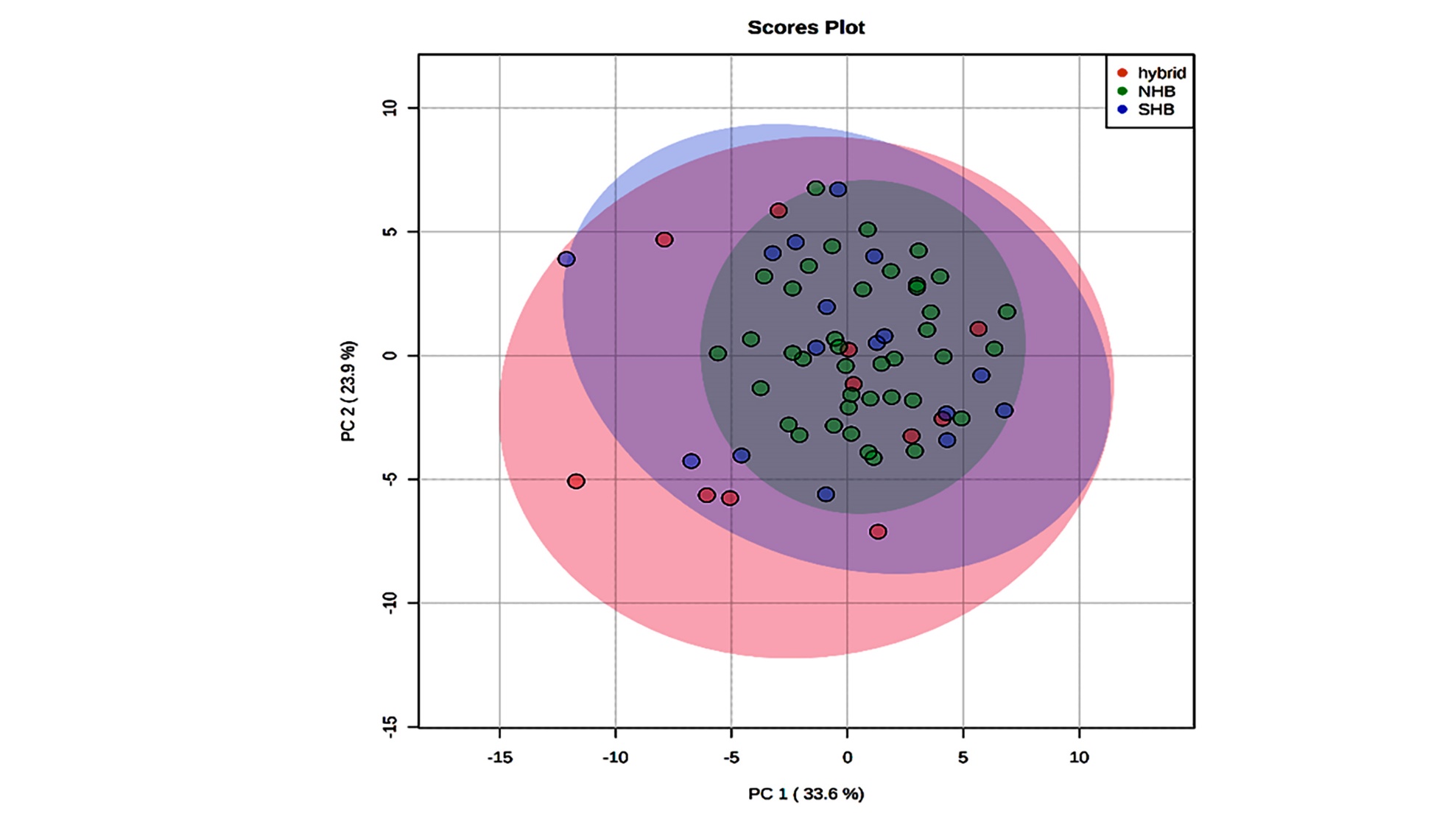


Supplementary Figure S7. PCA score plot of relative and absolute bioaccessibility and fruit quality data of 66 blueberry accessions based on blueberry classification of southern highbush (SHB), northern highbush (NHB) and hybrids.
